# Knowledge, attitude, and practices toward Hepatitis B infection among hemodialysis patients: A nationwide study in Jordan

**DOI:** 10.1371/journal.pone.0312226

**Published:** 2024-10-17

**Authors:** Nader Alaridah, Rahaf A. Jereisat, Sara Abu-Mutaw, Haneen O. Abuhani, Raba’a F. Jarrar, Rayan M. Joudeh, Basmalah Al-Hawadi, Saif Alhawadi, Razan Qasim Al-oyoun, Hasan Nassr, Mohammad Al-Taher, Bassel Qiqieh, Layan Ismail, Haneen Al-Abdallat, Anas H. A. Abu-Humaidan

**Affiliations:** 1 Department of Pathology, Microbiology, and Forensic Medicine, School of Medicine, University of Jordan, Amman, Jordan; 2 Princess Basma Comprehensive Health Center, Jordanian Ministry of Health, Amman, Jordan; 3 School of Medicine, University of Jordan, Amman, Jordan; 4 Faculty of Medicine, Al-Balqa Applied University, As-Salt, Jordan; 5 Department of Clinical Laboratory Sciences, School of Science, University of Jordan, Amman, Jordan; 6 Fellow, Ibn Sina University for Medical Sciences, Amman, Jordan; 7 King Abdullah University Hospital (KAUH), Jordan University of Science and Technology, Irbid, Jordan; 8 College of Medicine, Sulaiman Al-Rajhi University, Al-Bukayriah, Al-Qassim, Saudi Arabia; Al-Azhar University, EGYPT

## Abstract

The Hepatitis B virus (HBV) is a prevalent blood-borne illness, posing a significant risk to hemodialysis patients particularly due to their potential immunosuppressed status. This study aimed to address HBV awareness among Jordanian hemodialysis patients, filling a gap in regional research. A descriptive cross-sectional study was conducted at a multicenter governmental hospital in Jordan, with 389 participants. Among them, 61.3% were male, and 80.7% were over 38 years old. While 34% demonstrated a high level of knowledge, Participants with a higher degree of education and those working in the medical field were more informed. Although most participants had an inadequate understanding of HBV symptoms and transmission, they maintained positive attitudes and engaged in infection preventative actions. Enhanced educational efforts are required to raise awareness among hemodialysis patients, and further research is needed to address any reluctance towards preventive practices and seeking treatment.

## Introduction

Hepatitis B virus is a major clinical issue because of its high infectious rate and ability to proceed from acute liver damage to chronic liver disease [1]. It is considered one of the most ubiquitous bloodborne viruses globally, with around 468 million cases recorded in 2016 [2]. HBV prevalence is highest in the Western Pacific, followed by Africa [3]. In 2017, the estimated incidence of HBV in Jordan was about 2.4%, and the prevalence of HBV among patients in dialysis units was 6% [4, 5]. HBV-related liver transplants accounted for 17% of all liver transplants done at King Hussein Medical Center. [6] The incidence of HBV infection among dialysis patients in Western Europe, Japan, and the United States is around 6.6%, while it ranges from 1.3% to 14.6% in the Asia-Pacific region [7, 8]. Extrahepatic symptoms of HBV can be seen in both acute and chronic infections, affecting up to 20% of HBV-infected cases in Jordan [9]. It is generally understood that HBV is transferred during pregnancy and through contact with contaminated blood or bodily fluids [10]. In reality, hemodialysis patients are at increased risk of contracting HBV not only because of the transmission mechanism but also because of uremia-related immune weakness and dysregulation [11]. Clinical manifestations of HBV include acute hepatitis, chronic hepatitis, cirrhosis, hepatocellular carcinoma, hepatic failure, and other consequences [1]. In dialysis patients, liver disease has a discrete clinical course, moving from moderate hepatic inflammation to severe fibrosis [12]. Furthermore, HBV infection has been linked to an increased risk of hepatocellular diseases and a lower chance of kidney transplantation [13]. Researchers have extensively explored the relationship between HBV infection and glomerular diseases [14]. Recent study has demonstrated significant correlations between chronic hepatitis B and renal function impairment, as well as an increased chance of acquiring a low glomerular filtration rate (GFR) [14]. When determining HBV severity in dialysis patients, depending exclusively on liver enzymes such as serum ALT is inadequate since they do not necessarily correspond with disease severity. Furthermore, higher serum ALP levels can be linked to the related parathyroid Furthermore, there is little research on the relationship between viral loads and illness severity. Consequently, liver biopsy appears to be the only definitive approach to evaluate disease activity in dialysis patients [15]. Vaccination against HBV has shown to be efficient, producing 98% to 100% protection in the general population. Since its introduction in 1995, the HBV vaccine has resulted in a considerable drop in infection rates [6]. The most effective technique for providing immunological protection against HBV infection in dialysis patients is to provide the vaccination before they reach end-stage renal failure. This is because the reaction to the vaccination declines in proportion to the degree of renal damage [16]. A study conducted in Jordan in 2020 examined general knowledge of HBV found that public understanding of HBV was typically low to moderate. Awareness of factors such as disease prevalence, etiology, transmission, and high-risk populations was found to be low [6]. Therefore, this highlights the need to conduct further studies regarding *HBV* in the local population. Based on previous literature and the scarcity of local studies on this matter, this study aims to assess the awareness of *HBV* among hemodialysis patients in Jordan.

## Materials and methods

A cross-sectional descriptive study was conducted among hemodialysis patients in different Jordanian hospitals with hemodialysis units. Multiple governmental hospitals in Jordan that had dialysis units and one university-based hospital were included in the study; AL-Hussein / Salt Hospital, Al Basheer Hospitals, Princess Basma Hospital, AL-Nadeem Hospital, Al Zarqa Hospital, Prince Hamza Hospital and Jordan University Hospital. The data for this study were collected in the period between July and December 2022. Participants were recruited from lists provided by dialysis units, each list included all regular patients who had scheduled sessions for hemodialysis, then patients were approached before or after the session, according to their preferences if they were willing to participate in the study. The inclusion criteria for the study were as follows: 1) hemodialysis patients who were above 18 years of age. 2) patients who accepted to participate in the study. Those who met the inclusion criteria and agreed to participate were invited to complete a face-to-face interviews-based survey, after providing informed consent. A total of 398 hemodialysis patients participated in the study, which exceeds the minimum sample size required. The sample size calculation was based on a 5% marginal error and a 50% prevalence rate [17]. Exclusion criteria for the study included patients who were not conscious or unable to provide informed consent, patients below the age of 18, and patients who refused to complete the questionnaire.

### a). Questionnaire administration

The questionnaire was administered in an interview-based manner, containing 35 items, the survey was formulated based on a previously validated tool [18]. It consisted of four sections: in addition to the demographic data, a knowledge section with 20 questions, an attitude section with 7 questions, and a practices section with 8 questions. The questionnaire was then piloted with 30 respondents for its acceptability and consistency. Few modifications were needed after the pilot testing. Data from the pilot study was not included in the final analysis. The reliability (internal consistency) was assessed during the pilot study, Cronbach’s alpha level was 0.7 in the knowledge section. When the consistency and validity of the study questionnaire were stabilized, the instrument was made available for data collection.

### b). Ethical consideration

This study was conducted ethically following the World Medical Association Declaration of Helsinki. Written informed consent was obtained from participants to participate in the study and the study’s protocol was ethically reviewed by The Institutional Review Board (IRB) at AL Bashir Hospital, Amman, the Hashemite Kingdom of Jordan (reference number: M.B.A/3208) in meeting No. 2/2022.

### c). Statistical analysis

Data were extracted from a Google form, then extracted into an Excel sheet. Data cleaning was done to check for any missing data and outliers in the data set before processing into analysis. Descriptive statistics were analyzed and reported as either frequency and percentage or mean and standard deviation for each numerical and categorical variable, respectively. The Chi-square test was used to assess the relationship between demographic factors, knowledge, attitude, and practices. Multivariate regression analysis was used to evaluate each independent variable after controlling for possible confounders. Each correct response received one point. The cut-off-point to determine the good and poor level of knowledge was according to the bloom cut-off [19, 20]. If a participant correctly responded to 70% of the questions or more in each KAP section, the score was deemed to be good. If less than 70% of the questions in each section were correctly answered, the participant’s score was deemed poor. Microsoft Excel 2016 was used to enter the data, which was then imported into IBM SPSS version 25 (IBM Corp., Armonk, New York, USA) for analysis.

## Results

### Demographics of survey participants

The sociodemographic characteristics of participants are summarized in Table 1. Data was collected from 398 participants who agreed to be involved in the study. The percentage of male participants in our study was 61.3% (n = 244). Half of the participants were older than 50 years old 50% (n = 199), 30.7% (n = 122) were between 38–50 years old and the remaining 19.3% (n = 77) were between 18–37 years old. Almost four- fifths (n = 324) of participants were married. Regarding the educational level, (71.1%, n = 283) had a lower level of education. Around half of the participants (55.3%, n = 220) have no job. Only (3.3%, n = 13) were from the medical study field, (35.7%, n = 142) were non-medical. Almost three-fourths (n = 294) were city residents. The majority of participants (95.7%, n = 381) had no personal history of Hepatitis B infection, and (83.7%, n = 333) had no family history either.

**Table 1:** Demographics of survey participants.

Sociodemographic		n (%)
Gender	Female	154 (38.7%)
	Male	244 (61.3%)
Age	18–37	77 (19.3%)
	38–50	122 (30.7%)
	more than 50	199 (50%)
Marital Status	Single	74 (18.6%)
	married	324 (81.4%)
Education Level	Low	283 (71.1%)
	high	115 (28.9%)
Employment status	no work	220 (55.3%)
	work	178 (44.7%)
Study field	medical	13 (3.3%)
	non-medical	142 (35.7%)
	none	243 (61.0%)
Residence	city	294 (73.9%)
	village	104 (26.1%)
History of *HBV* in family	Yes	65 (16.3%)
	No	333 (83.7%)
History of *HBV*	Yes	17 (4.3%)
	No	381 (95.7%)

**Education level: High education means the participant has a diploma of education beyond high school

### Knowledge toward the Hepatitis B virus

Regarding participants’ knowledge about hepatitis B, results are stated in S1 Table in S1 Appendix. The majority of participants (85.4%, n = 340) had heard about hepatitis, with 60.3% (n = 240) particularly hearing about the hepatitis B subtype. However, almost half of them knew that it was a viral infection (52%, n = 207). Moreover, 70.9% (n = 282) knew that it affects the liver, but less than half of the participants (43.7%, n = 174) knew that liver cancer is a consequence of the infection. Despite the fact that only 29.4% (n = 117) knew that the initial symptoms of the infection could mimic a cold, 68.3% (n = 272) knew that jaundice is a common symptom of the infection. Speaking about symptoms, it is worth mentioning that only 37.9% (n = 151) knew that the infection could be asymptomatic, and 46.7% (n = 186) stated previous knowledge that nausea, vomiting, and anorexia are common symptoms. Participants’ knowledge about the routes of transmission was variable. 72.1% (n = 287) knew that it could be transmitted by unsterile syringes, needles and surgical equipment, 76.1% (n = 303) knew about contaminated blood and blood products, 59% (n = 235) knew about barber’s blades and nose piercing, 51% (n = 203) knew about unsafe sex and 48.2% (n = 192) knew about vertical transmission. However, only 21.4% (n = 85) knew that contaminated water/food prepared by an infected person is not a route of transmission. Regarding the management of hepatitis B, only 10.8% (n = 43) knew that it is not a curable disease, and 20.9% (n = 83) knew that it could be self-cured by the body. Moreover, 61.6% (n = 245) knew that there is a vaccine for the virus, but only 17.3% (n = 69) knew that no specific diet is required for the treatment.

### Attitudes toward the Hepatitis B virus

S2 Table in S1 Appendix summarizes participants’ attitudes answers toward hepatitis B infection. Almost, 74.9% (n = 298) thought that they could be infected with the virus, while the rest did not believe that they were susceptible to Hepatitis B infection. Regarding participants’ reactions in case they were infected, the majority 34.7% (n = 138) would be sad, 33.4% (n = 133) would be surprised, 30.2% (n = 120) would be afraid and only 1.8% (n = 7) would be ashamed. 67.8% (n = 270) would like to talk to physicians about their illness if they were infected, 14.8% (n = 59) would talk to their partners, 7.8% (n = 31) would talk to their relatives, 6% (n = 24) would talk to their parents and the minority 3.5% (n = 14) would talk to their children. 78.9% (n = 314) of the participants would go to doctors if they had symptoms of Hepatitis B, while 15.8% (n = 63) would go to a health facility, 2.3% (n = 9) would go to a traditional healer and the remaining 3% (n = 12) prefer not to go to any of these. Regarding the stage in which the participants decide to go to a healthy facility if they had symptoms, 78.9% (n = 314) said they would go as soon as they realized the symptoms, 13.1% (n = 52) would go after 3–4 weeks of realization, 5.3% (n = 21) would go if their treatment failed, 2.8% (n = 11) said they would not go at all. 5.7% (n = 182) of participants had no idea how expensive it would be to diagnose and treat hepatitis B, however almost a third 37.4% (n = 149) thought it would be expensive or somewhat expensive, 8.5% (n = 34) thought it would be free and 8.3% (n = 33) thought it would be reasonable.

### Practice toward Hepatitis B virus

As shown in S3 Table in S1 Appendix, 69.1% (n = 275) of participants had done the screening for hepatitis B in their lifetime, while the rest 30.9% (n = 123) had not. More than half of them 64.3% (n = 256) got vaccinated against hepatitis B. The majority of the participants 78.1% (n = 311) and 73.4% (n = 292) ask for a new syringe before use, and asked for screening of blood before transfusion respectively. When participants were asked if they asked their barber to use sterile tools that had not been used by others, 72.6% (n = 289) answered positively. However, the majority of participants 89.7% (n = 357) would do further investigations if they were infected while the rest would not. 62.8% (n = 250) avoid meeting patients with hepatitis B. A very small percentage 7.5% (n = 30) have participated in a program related to hepatitis B, whereas the majority 92.5% (n = 368) have not.

### Associated factors associated with knowledge level toward *HBV* infection

The significant associations between knowledge and sociodemographics are shown in Table 2. In the univariate logistic regression analysis, significant associations were found between the level of knowledge and the following variables: education level (OR = 1.888, CI = 1.207–2.954, p<0.005), work (OR = 1.772, CI = 1.165–2.694, p<0.007), medical study field (OR = 8.963, CI = 1.913–41.987, p<0.005), non-medical study field (OR = 0.659, CI = 0.426–1.022, p<0.062), history of hepatitis B virus infection in family (OR = 3.424, CI = 1.981–5.917, p<0.000). While in the multi variate logistic regression analysis, significant associations were found between the level of knowledge and the following variables: education level (OR = 1.490, CI = 0.878–2.528, p<0.140), work (OR = 1.589, CI = 0.819–3.082, p<0.171), medical study field (OR = 8.568, CI = 1.761–41.688, p<0.008), non-medical study field (OR = 0.959, CI = 0.467–1.970, p<0.909), history of hepatitis B virus infection in family (OR = 3.822, CI = 2.157–6.771, p<0.000), as shown in Table 3.

**Table 2:** Association between socio-demographic factors and knowledge level.

		Knowledge level				
		High knowledge		Low knowledge		
		Count	%	Count	%	P-value
Gender	Female	52	13.1%	102	25.6%	0.959
	Male	83	20.9%	161	40.5%	
Age	18–37	34	8.5%	43	10.8%	0.061
	38–50	34	8.5%	88	22.1%	
	more than 50	67	16.8%	132	33.2%	
Marital Status	Unmarried	32	8%	42	10.6%	0.060
	Married	103	25.9%	221	55.5%	
Education Level	Low	84	21.1%	199	50%	0.005*
	High	51	12.8%	64	16.1%	
Employment status	No work	62	15.6%	158	39.7%	0.007*
	Work	73	18.3%	105	26.4%	
Study field	medical	11	2.8%	2	0.5%	0.000*
	non-medical	54	13.6%	88	22.1%	
	none	70	17.6%	173	43.5%	
Residence	city	96	24.1%	198	49.7%	0.370
	village	39	9.8%	65	16.3%	
Family History of *HBV* infection	Yes	38	9.5%	27	6.8%	0.000*
	No	97	24.4%	236	59.3%	
Self-reported of *HBV* infection	Yes	9	2.3%	8	2%	0.090
	No	126	31.7%	255	64.1%	

(*) denote significance

**Table 3:** Logistic regression analysis between knowledge, and socio-demographics factor toward *HBV* infection.

Covariates	Knowledge					
	OR	CI	P-value*	OR	CI	P-value**
Education level:						
Low education	Reference			Reference		
Higher education	1.888	1.207–2.954	.005	1.490	.878–2.528	.140
Employment status						
No work	Reference			Reference		
Work	1.772	1.165–2.694	.007	1.589	.819–3.082	.171
Study field						
None	Reference			Reference		
Medical	8.963	1.913–41.987	.005	8.568	1.761–41.688	.008
Non-medical	.659	.426–1.022	.062	.959	.467–1.970	.909
Family History of *HBV* infection						
No	Reference			Reference		
Yes	3.424	1.981–5.917	.000	3.822	2.157–6.771	.000

*Univariate logistic regression.

**multi-logistic regression

## Discussion

Hepatitis B Virus (*HBV*) infection represents a global health challenge, affecting approximately 300 million individuals worldwide [1]. Among Hemodialysis patients, the risk of *HBV* infection is higher [16]. This risk stems from the frequent need for blood transfusions, the procedure itself, and the potential exposure to contaminated blood products and contact with healthcare personnel which favors infections in addition to their compromised immune status [21]. Thus, emphasizing the importance of increasing patient awareness regarding these risks. Implementing stringent infection control measures is a vital step in maintaining the health and well-being of hemodialysis patients. This hospital -based cross national study aimed to investigate the knowledge, attitude. and practices of hemodialysis patients towards hepatitis B virus. Data was gathered from a cohort of 398 participants, with a predominance of middle-aged to old individuals, and 61% were male participants. Notably, only 4.3% of the participants had reported a history of *HBV* infection.

Higher levels of knowledge and attitudes were observed among patients with higher educational levels, and among patients who already work in the medical field. On the contrary, a previous study reported limited knowledge among those with higher educational backgrounds [22]. The majority of the participants were already familiar with the term hepatitis, and 60% were aware of subtype B, with only half of the participants recognizing it as a viral infection. Furthermore, 70% of participants didn’t know that it can present early on with flu-like symptoms while more than half of them were aware that it presents with jaundice, nausea, and vomiting. The overall knowledge about potential consequences such as the risk of developing liver cancer was poor. Regarding transmission route, results were satisfactory in which majority of the participants know that it can be transmitted through the use of unsterilized syringes, surgical needles (72.1%) and blades (59%), and through blood and blood products (76.1%). This was correlated with what resulted in the practice assessment section where the majority followed protective measures against the transmission of *HBV* by these routes. However, half of the participants were unknowledgeable about its transmission through unsafe sexual intercourse (51%) and vertical transmission from mother to child (48.2%). Moreover, most participants (79.6%) have a misconception that having a drink or food from an infected person can increase the risk of acquiring the infection and this also can explain why two-thirds of participants avoid meeting with positive patients. In comparison to other studies, the level of knowledge of the disease presentation, transmission routes, and related complications, among the different populations were similar to our study [18, 23, 24]. Moreover, improving public and healthcare providers’ awareness of hepatitis B should be sought out by health policymakers to minimize discrimination against infected persons, reduce transmission, improve early detection and treatment, and prevent complications [25, 26]. In regards to attitudes, the majority of participants expressed a sense of vulnerability to infection (75%) and indicated that they would promptly seek medical attention (67.8%) if they suspected they were infected. Remarkably, half of the participants were unsure of the financial implications associated with the diagnosis and treatment of Hepatitis B, whereas 37.4% thought it would be expensive or somewhat expensive. Additionally, 62.3% expressed concern about the possibility of transmitting the infection to their families. These findings indicate that the majority of the participants are aware of the seriousness of the disease but lack sufficient knowledge and confidence in the treatment process or to address the disease’s impact on their daily lives, social interactions, and financial situation. Patients with chronic kidney disease must be vaccinated early, as their ability to achieve immunological protection with the *HBV* vaccine decreases with disease progression [27]. It is important to note that participants exhibited a positive result towards vaccination, with 61.6% being aware of the available vaccine, and 64.3% reported having already been vaccinated. Additionally, a majority expressed willingness to undergo further investigations and adhere to safety practices. However, 92.5% have never participated in *HBV*-related health education programs. In the quest for controlling HBV infections regionally and worldwide, lack of knowledge and understanding of HBV transmission dynamics among the public, highly susceptible groups, medical students, and healthcare professionals due to misconceptions in knowledge about HBV should be identified and further awareness must be spread and advocated [25, 26]. A similar cross-sectional study was done among Vietnamese Americans focused on *HBV* screening and vaccinations stating that only one third of the participants were aware of the availability of screening and vaccination against *HBV* [28]. Thus, implementing Community awareness campaigns, universal vaccination, targeted outreach programs for high-risk groups, patient education, and healthcare provider training can significantly decrease the spread of HBV and promote public health awareness in general.

### Strengths and limitations

The strength of our studies lies in that, this is the first paper assessing the KAP of *HBV* in hemodialysis patients. This is particularly significant as it shifts the focus to a high-risk group. On the contrary, the cross-sectional design of the study and reliance on self-reported data present a drawback. There may be under or over-reporting, and chances of social desirability bias cannot be overlooked. Further studies are needed to investigate the prevalence of hepatitis B infection among hemodialysis patients and to assess the long-term health outcomes of hepatitis B-infected hemodialysis patients, including the impact on their kidney functions, mortality rates, and overall quality of life. Additionally, further research is needed to explore the psychological and emotional impact of living with hepatitis B among hemodialysis patients detailing any associated stigma or social challenges, thereby offering a more holistic understanding of HBV infections in such highly susceptible groups and addressing any reluctance towards preventive practices and seeking treatment.

## Conclusion

In conclusion, overall knowledge of *HBV* and its symptoms and mode of transmission were deficient. On the contrary, participants showed positive attitudes and practices to mitigate disease transmission. More efforts should be taken to raise awareness of *HBV* and other transfusion-transmissible pathogens among hemodialysis patients. Additionally, more studies should be conducted to investigate patient awareness, knowledge, and behaviors related to infection control which could help guide interventions.

## Supporting information

S1 Appendix Respondents correct answers. (DOCX)

## Abbreviations

CI: Confidence interval
GFR: Glomerular filtration rate
*HBV*: Hepatitis B virus
IBM: International Business Machines
IRB: Institutional Review Board
OR: Odds ratio
SPSS: Statistical Package for Social Sciences
