# Supplementary material for: Knowledge, attitude, and practices toward Hepatitis B infection among hemodialysis patients: A nationwide study in Jordan
Source: PLoS One. 2024 Oct 17;19(10):e0312226. doi: 10.1371/journal.pone.0312226 (PMC11486397; doi:10.1371/journal.pone.0312226)
Supplement: S1 Appendix — (DOCX) [file pone.0312226.s001.docx]

Table 1: Knowledge questions

|  | Answer * | Count | % |
| --- | --- | --- | --- |
| Have you ever heard of disease termed hepatitis? | Yes | 340 | 85.4% |
| Have you ever heard of disease termed as hepatitis B? | Yes | 240 | 60.3% |
| Is hepatitis B a viral disease? | Yes | 207 | 52% |
| Can hepatitis B affect liver function? | Yes | 282 | 70.9% |
| Can hepatitis B cause liver cancer? | Yes | 174 | 43.7% |
| Can hepatitis B affect any age group ? | Yes | 257 | 64.6% |
| The early symptoms of hepatitis B are same like cold and flu fever running nose ? | Yes | 117 | 29.4% |
| Jaundice is one of the common symptoms of hepatitis B | Yes | 272 | 68.3% |
| Are nausea vomitting and loss of appetite common symptom of Hepatitis? | Yes | 186 | 46.7% |
| Are there no symptoms of the Hepatitis B in some of the patient? | Yes | 151 | 37.9% |
| Can Hepatitis B be transmitted by unsterilized syringes needles and surgic? | Yes | 287 | 72.1% |
| Can Hepatitis B be transmitted by contaminated blood and blood product? | Yes | 303 | 76.1% |
| Can Hepatitis B be transmitted by using blades of the barberear and nose piercing? | Yes | 235 | 59% |
| Can Hepatitis B be transmitted by unsafe sex? | Yes | 203 | 51% |
| Can Hepatitis B be transmitted from mother to child ? | Yes | 192 | 48.2% |
| Can Hepatitis B be transmitted by contaminated water/food prepared by person suffering with these infections? | No | 85 | 21.4% |
| Is Hepatitis B curable/treatable? | No | 43 | 10.8% |
| Can Hepatitis B be self-cured by body? | Yes | 83 | 20.9% |
| Is vaccination available for Hepatitis B? | Yes | 245 | 61.6% |
| Is specific diet is required for the treatment of Hepatitis B? | No | 69 | 17.3% |

*Correct Answers

| Table 2: Attitudes and questions | Frequency | % |
| --- | --- | --- |
| Do you think you can get hepatitis B? | | |
| No | 100 | 25.1% |
| Yes* | 298 | 74.9% |
| What would be your reaction if you found that you have hepatitis B? | | |
| Fear* | 120 | 30.2% |
| Shame | 7 | 1.8% |
| Surprise | 133 | 33.4% |
| Sadness | 138 | 34.7% |
| Who would you talk to about your illness? | | |
| Physician | 270 | 67.8% |
| Spouse | 59 | 14.8% |
| Parents | 24 | 6% |
| Child | 14 | 3.5% |
| Other relatives | 31 | 7.8% |
| What will you do if you think that you have symptoms of hepatitis B? | | |
| Go to the health facility* | 63 | 15.8% |
| Go to doctor | 314 | 78.9% |
| Go to traditional healer | 9 | 2.3% |
| Will not go to physician | 12 | 3% |
| If you had symptoms of Hepatitis B, at what stage you will go to the health facility? | | |
| Own treatment fails | 21 | 5.3% |
| After 3-4 weeks of the appearance of symptoms are of Hepatitis B | 52 | 13.1% |
| Soon as I realize the symptoms are of Hepatitis B* | 314 | 78.9% |
| Will not go to physician | 11 | 2.8% |
| How expensive do you think is the diagnosis and treatment of Hepatitis B? | | |
| Free | 34 | 8.5% |
| Reasonable | 33 | 8.3% |
| Somewhat expensive | 65 | 16.3% |
| Expensive | 84 | 21.1% |
| Don't know* | 182 | 45.7% |
| What worries you most if you will be diagnosed with Hepatitis B? | | |
| Fear of death | 84 | 21.1% |
| Fear of disease spread to family | 248 | 62.3% |
| Cost of treatment | 30 | 7.5% |
| Isolation from the society* | 36 | 9% |

*Answers that indicate good attitude

| Table 3: Practice questions and answers | | Count | % |
| --- | --- | --- | --- |
| Have you done screening for Hepatitis B | No | 123 | 30.9% |
|  | Yes* | 275 | 69.1% |
| Have you got yourself vaccinated against Hepatitis B | No | 142 | 35.7% |
|  | Yes* | 256 | 64.3% |
| Do you ask for a new syringe before use | No | 87 | 21.9% |
|  | Yes* | 311 | 78.1% |
| Do you ask for screening of blood before transfusion | No | 106 | 26.6% |
|  | Yes* | 292 | 73.4% |
| Do you ask your barber to change blade Or for safe equipment's for ear and nose | No | 109 | 27.4% |
|  | Yes* | 289 | 72.6% |
| In case you are diagnosed with Hepatitis B would you go for further investing a | No | 41 | 10.3% |
|  | Yes* | 357 | 89.7% |
| Do you avoid meeting Hepatitis B patients | No | 148 | 37.2% |
|  | Yes* | 250 | 62.8% |
| Have you ever participated in health education program related to Hepatitis | No | 368 | 92.5% |
|  | Yes* | 30 | 7.5% |

**Answers that indicate good Practices
